# Lactating Mother and Psychotropic Drugs

**DOI:** 10.4103/0973-1229.58821

**Published:** 2010

**Authors:** B. M. Tripathi, Pradipta Majumder

**Affiliations:** * *Professor, Department of Psychiatry and National Drug Dependence and Treatment center, All India Institute of Medical Sciences, New Delhi, India*; ** *Resident, Department of Psychiatry, All India Institute of Medical Sciences, New Delhi - 110029, India*

**Keywords:** *Lactation*, *Psychotropics*, *SSRIs*, *Antipsychotics*, *Mood stabilizers*

## Abstract

Usage of psychotropics during pregnancy and lactation has always been a topic of debate and controversy. The debate stems from the potential adverse effects on the growing fetus or infants due to the transfer of psychotropic drugs through placenta or breast milk of mothers receiving them; and the problem of discontinuing psychotropics in lactating mother considering chances of relapse. However, most of the psychotropics are found to be relatively safe when used cautiously during the lactation phase. This article describes available data on the use of psychotropics in lactating mothers, in particular, in relation to the safety profile of infants.

## Introduction

Pregnancy is a period of great turmoil in terms of mental and physiological health. It is accompanied by a role transition from womanhood to motherhood and partly due to this; pregnancy and puerperium often increase the chance of development or exacerbation of psychiatric illnesses.

Clinical depression, for example, occurs in 10–15% of women during pregnancy, while postpartum depression occurs in around 10–22% of women (Kim, 2006). Women most at risk for developing depression have a psychiatric history of recurrent depressive episodes or prior postpartum depression (Misri, 2000). It is also increasingly recognized that some women diagnosed with depression during postnatal phase have been depressed during the antenatal period as well. So it is important to detect and treat symptoms of depression in the antenatal period to reduce the incidence of post-natal depression (PND) (Louise, 2006). More than 50% of women who discontinue antidepressants need them again later in pregnancy because of a resurgence of depressive symptoms (Misri, 2000). In women with moderate and severe depressive disorders, it is being found that drug treatment during pregnancy and in the postnatal period has significant benefits for both mother and infant (Kristensen, 2007). The potential negative consequences of post-natal depression on mother as well as child have been well established in the literature. The negative consequences of maternal depression on the cognitive, motor and emotional development of the offspring have already been discussed much in the earlier literature (Kim, 2006). However, it remains a concern in prescribing antidepressants as well as other psychotropics during pregnancy as well as during lactation due to the fear of transferring psychotropics to the infants through placenta or breast milk.

A statement on the transfer of drugs and chemicals into human milk was first published in 1983, with revisions in 1989 and 1994 (American Academy of Pediatrics, 2001). There is particular concern regarding prescribing antidepressants when women are breast-feeding, as there is limited evidence on the extent to which infants are exposed to antidepressants through breast milk and the long-term effect this may have on them (Howard, 2005). Despite this, in the United States, 2.8% of pregnant women reported the use of a serotonin reuptake inhibitor (SRI) in the three months preconception and during gestation. With four million pregnancies that result in live births, more than 90,000 women will be exposed to an SRI yearly (Dorothy, 2008).

On an average, nursing mothers produce 600 to 1,000 ml of milk daily (Burt, 2001). Most drugs are transferred into milk by the passive diffusion processes and hence maternal drug concentration (controlled by maternal pharmacokinetics) and M/P (milk plasma ratio) are major determinants of infant dose via milk (Rampono, 2006). Active or carrier-mediated transport occurs for some agents. Drugs must pass from the maternal plasma, through the capillary walls, into the alveolar cells lining milk duct. During the first few days of life there are large gaps between these alveolar cells, which allow most molecules to cross through easily. Once mature milk is established, these gaps are closed and drug access to the milk is more limited (Moretti, 2009).

The passage of psychotropic medications through breast milk depends upon several factors including administration route, absorption rate, half-life, and time of peak serum concentration, dissociation constant, volume of distribution, molecular size, degree of ionization, pH of plasma and milk, solubility of drugs in water and lipids, and greater binding to plasma protein than to milk protein (Gentile, 2004). The higher lipid content of hind milk (the milk ejected during the second half of a feeding) makes it likely that the second half will have a higher concentration of maternal medication than the first half (fore-milk) (Burt, 2001).

Though there is some evidence of transfer of these psychotropics through breast milk and there are some concerns regarding the potential adverse consequences on the baby, the rational solution to this problem could not be to just stopping breast feeding the baby and continue psychotropics to safeguard mothers’ interest without taking into account the specific advantages of breast feeding. The World Health Organization (WHO), the American Academy of Pediatrics (AAP) and the American College of Obstetricians and Gynecologists recommend breast milk exclusively for at least the first six months of life and continued breast milk with food through 6–12 months of age. There are evidences for significant health-related, nutritional, immunologic, developmental, psychological, social, economic, and environmental advantages for breastfeeding (Becker, 2009). Also, studies have shown that the fertility rates among women suffering from psychotic disorders are at a lower range than that of the general population, partly because of the hyperprolactinemia secondary to the prescribed antipsychotic medications (Howard, 2004), which make their pregnancies more precious, and interest and well-being of the baby cannot be overlooked (Gardiner, 2003). This means, the baby should get the benefits of breast-feeding as a part of her normal growth and development. Due to these reasons, it might be important for specialized units to deal with post-natal psychiatric illnesses, yet units devoted to post-partum care only exist in a few countries (Berle, 2004).

Considering these, it is often being emphasized that treatment of psychiatric illnesses in women after childbirth should integrate both psychosocial and biological modalities in order to minimize the risk of affection of the baby due to exposure to psychotropics prescribed to the mother (Berle, 2004). It should be carefully considered that psychotropics are usually given on a long-term basis, unlike antibiotics and some other medications. Therefore, it also achieves a steady state concentration in the excreted milk and there is little use in discarding breast milk, or timing feeding around medication as it is thought to make little difference to the dose received by the infant (Snellen, 2007).

Almost all the psychotropics are excreted in breast milk and have the potential to cause poor feeding, sedation and other side effects in infants. Besides, as a matter of fact, premature infants and infants less than six months of age have a limited capacity to metabolize these drugs due to the immature hepatic function. There is a notional safety limit of a relative infant dose of less than 10% of the maternal dose, which can be used to understand data around breast milk excretion (Snellen, 2007).

Because of these concerns, the US FDA has categorized drugs on the basis of their safety during pregnancy. However, the letter system which categorizes drugs into risk categories A, B, C, D and X is going to be eliminated as per the recent US FDA announcement (May, 2008). This system is considered outdated and does not account for new information about drug safety and risk profiles in pregnancy and lactation. Additionally, it is being criticized as being over-simplified. The new labeling system will have separate sections for pregnancy and for lactation, and each section will have three main components: risk summary, clinical considerations, and analysis of data (animal vs. human) (Becker, 2009).

The following paragraphs would try to summarize the usage of psychotropics during lactation, mainly in terms of safety among infants. We would like to describe the available data by dividing it in different group of psychotropics.

### SSRIs

Most SSRIs and SNRIs are found in milk in relatively low amounts and are considered “probably safe” in treating depression as well as post-partum dysthymia, panic disorder and obsessive-compulsive disorder; and often considered the first line agent for these disorders (Berle, 2004). Fluoxetine as well as other SSRIs, although used frequently in pregnancy as well as in lactation, are secreted in breast milk and the infant dose is largely determined by the concentration of the maternal drug (Kim, 2006). Neonatal withdrawal syndrome is repeatedly reported with late-trimester use of fluoxetine, sertraline, and paroxetine (Misri, 2000).

Of some concern is a single case of a six-week-old infant of a mother receiving fluoxetine and developing excessive cry, decreased sleep, vomiting, and diarrhea that dissipated upon discontinuation of nursing. Adverse effects reported in other studies were transient and by maternal report or were confounded by multiple medications (Burt, 2001).

Two case reports of nursing babies exposed to milk from mothers taking fluoxetine reported increased irritability, colic, increased crying, decreased sleep, increased vomiting, and watery stools (Isenberg, 1990; Lester, 1993).

Another case report found substantial concentrations of both fluoxetine and norfluoxetine in infants’ serum and reports of seizure like activity (Misri, 2000). In most studies, no significant adverse consequences were documented with the use of Fluoxetine in lactating mothers up to one year of their children. Fluoxetine 20–40 mg resulted in relatively low infant plasma levels (Becker, 2009).

With paroxetine and sertraline, two of the more commonly used agents; infants generally had low or undetectable serum drug levels (Moretti, 2009). Contradictory evidence colors the safety of sertraline in nursing mothers although most of the literature suggests excretion of the drug in breast milk. There remains a concentration gradient in excretion of sertraline in human breast milk, such that the highest concentration exists in the hind milk 7-10 hours of maternal dosage (Misri, 2000). However, most of the literature did not point towards adverse consequences following its use (Becker, 2009).

The data on other SSRIs did not show much concern regarding its use among lactating mothers since in most of the studies the drug level among infants were low to undetectable (Becker, 2009). Relative infant doses of SSRIs have been found to be as follows: Sertraline 2.2%, Citalopram& Escitalopram 3.6%, Paroxetine 2.1%, Fluvoxamine 1.3%, Fluoxetine 6.8% (Snellen, 2007).

Based on extensive research, breast-feeding should not be generally discouraged in women using SSRIs. There is still, however, a lack of long-term data concerning infant antidepressant drug exposure through breast milk (Berle, 2004).

## Tricyclic Antidepressants

Tricyclics have a less favorable side effect profile and a much higher risk of morbidity and mortality from overdose. However, it is relatively safer and low levels of drugs are secreted for most tricyclics (Snellen, 2007). In most cases, there have been no adverse effects found with exposure to nortriptyline, imipramine, desipramine, or clomipramine (Becker, 2009). Maternal doxepine use during pregnancy and lactation has raised some concern.

Two case reports mention respiratory depression, drowsiness, hypotonia, poor suck, vomiting in infants of mother receiving doxepine. In both these reports the infant blood concentration levels of doxepine were low and both the reported infants recovered following 24-48 hours of discontinuation of breast feeding (Matheson, 1985; Frey, 1999). Authors advise to avoid doxepine if possible due to reported risk of dangerous sedation, respiratory arrest and poor sucking (Snellen, 2007; Becker, 2009).

However, if the patient has had good improvement in the past, there is less logic in changing the medication during lactation (Moretti, 2009). Relative infant doses of the TCAs may be as follows: Amitryptiline 1.5%, Clomipramine 2.8%, Dothiepin 4.4%, Doxepin 1.2%, Imipramine 0.15%, and Nortriptyline 1.5%. It is necessary to monitor all infants closely for sedation (Snellen, 2007).

## Mirtazapine, Venlafaxine, Mianserin, Reboxetine & Bupropion

Little evidence exists for all except Venlafaxine and Mirtazapine. Studies for these are compromised by small sample size, thereby decreasing the generalizability of drawing reliable conclusions. Relative infant doses of Venlafaxine and Mirtazapine were found to be around 6.4% and 1.9% respectively (Snellen, 2007). It has been mentioned in certain studies that Mirtazapine can be used as first-line treatment and, because of its action on histamine H_1_ receptors, may be preferred in some patients with postnatal depression, when night-time sedation is required (Kristensen, 2007). Though there is transfer of Mirtazapine in the breast milk of the lactating mother, the infant dose is much less than that considered safe. In a recent study by Kristensen (2007), it was found that the calculated mean relative infant dose for Mirtazapine plus its desmethyl metabolite was some 1.9% (as Mirtazapine equivalents) of the weight-adjusted maternal dose which is much less than the limit of 10% often mentioned as safe. In the same study, the calculated relative infant dose of escitalopram plus desmethylescitalopram was 4% (data of which was not shown in detail). Amongst the few available studies addressing use of Bupropion during lactation, no adverse consequences were documented except in one case report which documents the development of seizure in a six-month-old infant, which was possibly due to the use of Bupropion during breast feeding (Becker, 2009). No studies could be found on the use of Duloxetine during lactation.

## Mood Stabilizers

The AAP Committee on Drug Safety considers lithium to be associated “with significant effects on some nursing infants and should be given to nursing mothers with caution” (Becker, 2009). There are potential hazards as breast milk contains 30-50% of maternal serum level of administered lithium given the fact that neonates are far more sensitive to the adverse consequences of lithium than adults. Moreover, administered lithium usually does not reach steady state until day 10. Hence, effects are not seen in the early peri-partum period (Snellen, 2007). In the few case reports, adverse infant effects reported have included cyanosis, hypotonia, heart murmur, electrocardiographic changes, lethargy, and hypothermia. Infants may be more susceptible to both dehydration and lithium toxicity due to their immature kidney function and the potential for rapid dehydration. Therefore, the hydration status, BUN and creatine, lithium level, and thyroid levels should be carefully monitored in both mother and baby if it is necessary to use lithium in nursing mothers (Sivertz, 2005; Becker, 2009). It has been also recommended by some authors to use lithium with caution, and only if no other options are available; and the infant must be referred to a pediatrician for regular monitoring of lithium levels and thyroid function (Snellen, 2007).

Since most authors agree that the amount of valproic acid transferring to the infant via milk is low, breastfeeding while receiving this medication appears to be safe. Relative infant dose for valproate ranges from 0.68% to 7.6% (Becker, 2009). Although valproic acid may be effective and safer than lithium for breastfeeding mothers, at least three studies suggest that even lithium, when monitored closely, appears relatively safe, but great caution is recommended (Hale, 2004). Only one adverse event of thrombocytopenia and anemia in an exposed 3 month old infant was reported (Becker, 2009; Burt, 2001). Due to this, the infant should be monitored closely for liver function and platelet changes (Snellen, 2007).

In case of carbamazepine use, the levels reported in infant serum were highly variable, from 15% to 65% of maternal levels (Becker, 2009). Relative infant dose in case of carbamazepine is around 4.35% (Snellen, 2007) and is considered compatible with breast-feeding. In a review, it was found that only two of the 25 cases of carbamazepine exposure in nursing infants developed transient hepatic dysfunction (Burt, 2001). Still, exposed infants should be monitored by serum levels and liver function tests (Becker, 2009).

Both carbamazepine and valproic acid have not been associated with significant adverse events in breast-fed infants as small amounts are detectable in milk. They are considered compatible with lactation (Moretti, 2009).

The effects of lamotrigine are classified by the AAP as “unknown, but may be of concern” (Becker, 2009). In practice, it is currently considered moderately safe. However, there remains a theoretical concern about development of Stevens Johnson Syndrome and potentially life threatening rash amongst infants exposed to the drug through breast milk. However, none of the case reports found adverse effects in infants (Becker, 2009). It is advised to breast feed the baby with caution due to high milk: serum ratio of the excreted drug. It is found that relative infant dose is 22.7- 30% (Snellen, 2007).

Gabapentin has been reported to cross into breast milk at almost 100% of the maternal levels and is, therefore, not recommended for use in breastfeeding women (Sivertz, 2005).

**Table 1:** Summary of effect of exposure of psychotropics during lactation

Drugs	Effects observed
TCAs	Can be used with moderate safety
	Respiratory depression and over sedation have been documented in infants of mothers receiving Doxepine
SSRIs	Can be used with moderate safety
Benzodiazepines	Sedation, lethargy, withdrawal have been documented among infants after prolonged use
Antipsychotics	Recent data on haloperidol, olanzapine, quetiapine are encouraging Clozapine to be avoided as far as possible
	Still there is dearth of data regarding the safety of antipsychotics
Mood stabilizers	Valproate and carbamazepine are preferred. Lithium to be avoided as far as possible

Note: Adapted from Sivertz *et al* (2005).

## Antipsychotic Medication

Around 2% of women develop a non-affective psychotic disorder, and more than half of them have children (Howard, 2004). In a meta-analysis Altshuler *et al*. (1996) reported that exposure to low potency antipsychotics during the first trimester was associated with a small additional risk of congenital anomalies (Yaeger, 2006). The typical antipsychotics and clozapine are rated by the AAP as “unknown and may be of concern” to nursing infants (Becker, 2009) whereas, the atypical antipsychotics are mostly not rated till date due to very small bulk of literature to comment upon their safety. The available case reports in case of use of atypical antipsychotics mention low to undetectable drug level among infants of mothers using atypical antipsychotics. Some excretion occurs in all anti-psychotic medication, however the available data to date suggest that levels are low (generally < 3% of maternal dose) (Moretti, 2009).

Once again there is more evidence to suggest moderate safety with typical when compared with atypical anti-psychotics due to an extreme lack of data in the latter group. Moreover, longitudinal data is lacking for all antipsychotics. Again, literature support is too minimal to comment upon (Becker, 2009). There are a few cases of increased sedation in breast fed infants exposed to chlorpromazine and it is advisable to observe for sedation (esp. poor feeding) if used (Snellen, 2007).

**Figure 1:**
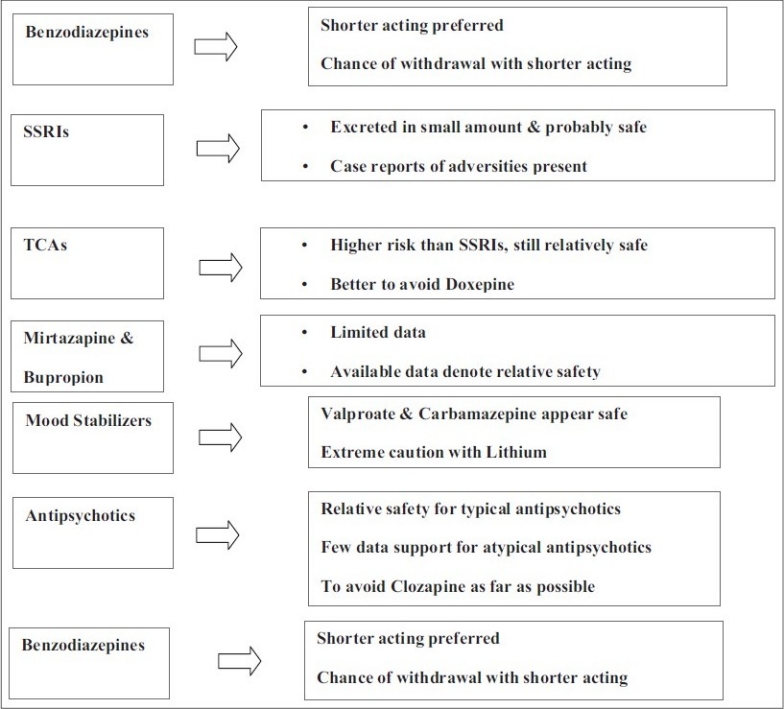
Flowchart of Paper

Literature also mentions that the use of phenothiazine sedatives such as promethazine or chlorpromazine has been reported to induce sleep apnea and may increase the risk of sudden infant death syndrome (SIDS). Some authors also advise to avoid these drugs in breastfeeding mothers if possible during the first six months postpartum when the risk of SIDS is highest. Thereafter, the infant should be observed closely for any sedative effects (Hale, 2004).

A case report mentioned developmental delay in case of babies exposed to haloperidol and chlorpromazine at 12-18 months of age (Wright, 1991). There is a risk of agranulocytosis, sedation and cardiovascular instability with clozapine use during lactation (Gentile, 2004). Breast milk excretion has varied in studies -- some indicate low levels others have shown high concentrations (Sivertz, 2005). Some authors advocate that clozapine recipient mothers should not breast feed their children in order to prevent the potential untoward effect on babies’ granulocyte count (Howard, 2004).

On comparing case registry data (n = 37) with historical controls the rates of adverse effects with olanzapine during pregnancy were not found to be higher (Goldstein, 2000). However, data from the University of Newcastle upon Tyne, NHS, (2009), National Teratology Information Service indicate an increased incidence of malformations of 10% (compared with an expected incidence of 2-3%). A small study of Quetiapine found levels not detectable in breast milk in doses less than 75mg. Small studies on breast milk excretion in olanzapine and risperidone suggest levels <5% (Snellen, 2007).

Limited data on olanzapine denote that the median and maximum relative infant doses is around 1.0% and 1.2% which indicate that infant exposure to olanzapine through milk is much less than the notional cutoff of 10.0% that has been used to guide drug safety during breast-feeding. It is also being recommended in certain studies that mothers trying to minimize infant exposure may wish to avoid breast-feeding at peak milk concentrations of the drug (i.e., five hours postdose) (Gardiner, 2003). However, a recent review mentions that use of olanzapine during lactation seems to be associated with an increased risk of inducing extrapyramidal reactions in the breast-fed babies and as such due care should be taken (Gentile, 2008). Among 21 reports of breast-fed infants exposed to olanzapine, five showed adverse events including jaundice, sedation, cardiomegaly, and heart murmur; shaking, poor suckling, and lethargy; protruded tongue; and rash, diarrhea, and sleeping disorders (Gentile, 2004).

## Benzodiazepines

There is little data on the safety of benzodiazepine during breast-feeding. However, benzodiazepines are excreted minimally in breast milk, e.g. 1% with oxazepam, 8% with alprazolam (Moretti, 2009). Generally, the evidence shows that benzodiazepines have lower infant milk/plasma ratios than other psychotropic medications. Benzodiazepines with shorter half-lives (i.e., lorazepam, alprazolam, and oxazepam) have been found to be very low in breast milk. No adverse effects were found in most exposed infants (Becker, 2009). Case reports indicate that milk plasma concentrations vary from 0.1 to 0.5% of the maternal dose for different benzodiazepines (Sivertz, 2005). Shorter acting agents such as oxazepam, alprazolam and temazepam are preferred by most authors. Moreover, there is risk of accumulation of these drugs in the infants’ body with longer acting agents. In an older study by Fisher (1985) only one of the 13 infants of lactating mothers receiving clonazepam developed cyanosis at the time of delivery and during the initial 10 days of lactation.

However, in view of certain case reports documenting the withdrawal effects on neonates of lactating mothers taking benzodiazepines, especially those who use benzodiazepines for the long term, it is often advocated to restrict use of benzodiazepines on a short-term basis (1 to 2 wk) (i.e., diazepam, midazolam, or lorazepam) rather than long term use, as far as possible (Hale, 2004). It is also being suggested to observe for sedation in the infant (Snellen, 2007).

### Zolpidem& Zopiclone

It is advised that these drugs be avoided due to availability of limited safety data on their usage. If used, observation of the infants for sedation is warranted (Snellen, 2007).

## Concluding Remarks

There are concerns regarding the use of psychotropics in lactating mothers in terms of their safety in infants. However, there is a paucity of quality researches addressing this issue which often warrants clinical challenge in day-to-day practice. The available evidence base to date suggests that psychotropic drugs as a group are relatively safe during pregnancy and lactation, and women and their healthcare providers should not be unduly concerned if a woman requires treatment. The clinician should be cautious about the uncommon but potential hazardous consequences observed among infants. Though there is evidence of transfer of most psychotropic medications through breast milk, drug concentration in the infants in most instances is less than the safety limit. However, there are instances of development of withdrawal symptoms and some other untoward effects observed among infants of lactating mothers receiving psychotropics, which need to be closely monitored.

### Take home message

During lactation, SSRIs can be used with moderate safety. Among mood stabilizers, valproate and carbamazepine are considered safe. Regarding antipsychotics, whenever possible, use of clozapine should be restricted. However, considering the limited evidence base further researches with good methodology are required to draw a conclusion in regard their use during lactation.

### Conflict of interest

None declared.

### Declaration

This is our original unpublished work, not submitted for publication elsewhere.

## Questions That Paper Raises

1. Will clinical features or infant serum level guide the decision of continuing psychotropics during lactation?
2. Should psychotropics with greater propensity to cause adverse consequences in infants be changed to safer alternatives even if they have shown good response in treating maternal mental disorders?
3. Can psychosocial interventions substitute for psychotropic medications during lactation?
4. Is it safer to opt for repeated transmagnetic stimulation, vagal nerve stimulation or modified ECT rather than pharmacotherapy during lactation?
5. Can Indian mothers afford withholding breast-feeding the infants given the fact of poor socio economic status and malnutrition of many sections of the Indian population?

## About the Author


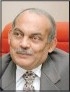
 *B. M. Tripathi, M.D. (Psychiatry) MRC Psych. (U.K), is Professor, Department of Psychiatry and National Drug Dependence and Treatment Center, All India Institute of Medical Sciences, New Delhi. Besides being an eminent teacher he has several publications in different national and international journals. He is also involved with various WHO-sponsored projects.*

Email id: bmt_54@yahoo.com


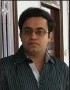
 *Pradipta Majumder is a final year resident of the Department of Psychiatry, All India Institute of Medical Sciences, New Delhi. He has passed MBBS from Calcutta Medical College, Kolkata, India. Besides taking keen interest in different academic activities in the department he also takes active participation in different social works organized by the department.*

Email drpradipta@yahoo.co.in
